# Comparison of Clinical Outcomes of Transverse Myelitis Among Adults With Myelin Oligodendrocyte Glycoprotein Antibody vs Aquaporin-4 Antibody Disease

**DOI:** 10.1001/jamanetworkopen.2019.12732

**Published:** 2019-10-09

**Authors:** Romina Mariano, Silvia Messina, Kurun Kumar, Wilhelm Kuker, Maria Isabel Leite, Jacqueline Palace

**Affiliations:** 1 Nuffield Department of Clinical Neurosciences, University of Oxford, Oxford, United Kingdom; 2 Department of Neuroradiology, Oxford University Hospitals National Health Service Trust, Oxford, United Kingdom

## Abstract

**Importance:**

Recognizing the differences between transverse myelitis (TM) associated with myelin oligodendrocyte glycoprotein (MOG) antibody (Ab) disease vs aquaporin-4 (AQP4)-Ab disease and prognosticating patients within each group may be an important factor for better clinical treatment for these respective patients.

**Objectives:**

To compare the clinical and radiological findings of the first TM episode in patients with MOG-Ab disease vs patients with AQP4-Ab disease and to assess factors associated with worse outcomes and relapse risk.

**Design, Setting, and Participants:**

This retrospective cross-sectional study used data collected from the Oxford Neuromyelitis Optica Service database, a national service that serves the south of England, including detailed clinical data, and high-quality imaging from within 4 weeks of the first TM episode from patients with MOG-Ab disease or AQP4-Ab disease and a confirmed history of TM from April 2018 to January 2019. Data analyses were conducted from February 2019 to April 2019.

**Main Outcomes and Measures:**

Onset features of each condition measured using the Expanded Disability Status Scale (EDSS) score, time to an EDSS score of 6, time to relapse, and residual sphincter dysfunction at least 6 months after the first TM episode and at last follow-up.

**Results:**

The total cohort included 115 adult patients, including 46 patients with MOG-Ab disease and 69 patients with AQP4-Ab disease. Patients with AQP4-Ab disease, compared with patients with MOG-Ab disease, tended to be older at onset of disease (mean [SD] age, 48.5 [14.9] years vs 33.7 [1.2] years) and female (57 [83%] women vs 24 [52%] women). Transverse myelitis occurred at onset of disease for 32 patients (70%) with MOG-Ab disease and 57 patients (78%) with AQP4-Ab disease. Onset severity did not differ between groups. An acute disseminated encephalomyelitis–like presentation occurred at the time of the TM in 4 patients (9%) with MOG-Ab disease but no patients with AQP4-Ab disease. Compared with patients with AQP4-Ab disease, patients with MOG-Ab disease were more likely to have short cord lesions (22 patients [48%] vs 10 patients [15%]; *P* < .001) and multiple cord lesions (18 patients [39%] vs 7 patients [10%]; *P* < .001). Approximately 50% of patients with MOG-Ab disease had only short cord lesions when the TM occurred as a relapse. Median (range) recovery EDSS score was lower in patients with MOG-Ab disease than patients with AQP4-Ab disease (1.8 [1.0-8.0] vs 3.0 [1.0-8.0]). Persistent bladder dysfunction associated with an increased prevalence of conus lesions occurred more frequently in patients with MOG-Ab disease than in patients with AQP4-Ab disease (27 patients [59%] vs 33 patients [48%]). Long-term catheter requirement was roughly equal between groups (9 patients [20%] vs 16 patients [23%]). Relapses after TM occurred in 17 patients with MOG-Ab disease (37%) and 36 patients with AQP4-Ab disease (52%). Concomitant brainstem lesions in patients with MOG-Ab disease were associated with a higher mean (SD) EDSS score at recovery (3.5 [2.3] vs 1.4 [0.9]; *P* < .001). In patients with AQP4-Ab disease, those younger than 50 years were more likely to relapse (27 of 36 patients aged <50 years [75%] vs 9 of 33 patients aged ≥50 years [27%]; *P* < .001) and those 50 years and older were more likely to reach an EDSS score of 6 (19 of 33 patients aged ≥50 years [58%] vs 11 of 36 patients aged <50 years [31%]; *P* = .03).

**Conclusions and Relevance:**

This study found that in patients who experienced a TM episode, short and multiple lesions at onset were more common in those with MOG-Ab disease than among those with AQP4-Ab disease. The presence of a brainstem lesion at the time of a TM episode in patients with MOG-Ab disease was associated with a worse recovery. In patients with AQP4-Ab disease, those 50 years and older at disease onset had more disability, and those younger than 50 years at disease onset had more relapses.

## Key Points

**Question:**

What clinical and radiological outcomes are associated with transverse myelitis in patients with myelin oligodendrocyte glycoprotein (MOG) antibody (Ab) disease or aquaporin-4 (AQP4)-Ab disease?

**Findings:**

In this cross-sectional study of 115 adults with MOG-Ab disease or AQP4-Ab disease, overall mobility recovery was better in patients with MOG-Ab disease, but sphincter dysfunction remained a significant feature. Having a concomitant brainstem lesion was associated with a worse prognosis in patients with MOG-Ab, whereas worse outcomes in patients with AQP4-Ab were associated with older age at onset of the disease.

**Meaning:**

Recognizing the differences between TM associated with MOG-Ab disease vs AQP4-Ab disease may be associated with better risk stratification of patients within each group.

## Teaser

This cross-sectional study examines clinical and radiological outcomes among patients with myelin oligodendrocyte glycoprotein (MOG) antibody (Ab) disease or aquaporin-4 (AQP4)-Ab disease after experiencing a first transverse myelitis episode.

## Introduction

Neuromyelitis optica spectrum disorder (NMOSD) is an autoimmune inflammatory disorder of the central nervous system characterized by episodes of optic neuritis (ON) or transverse myelitis (TM). Aquaporin-4 (AQP4) antibodies (Abs) are a highly specific pathologic marker of NMOSD and associated with a chronic relapsing condition with high morbidity and mortality.^1^ The discovery of myelin oligodendrocyte glycoprotein (MOG)–Ab disease has suggested that MOG-Ab disease is clinically and radiologically similar to NMOSD.^2^ In both conditions, TM is a hallmark feature, and disability appears to be associated with TM episodes. A 2017 study^3^ suggested that MOG-Ab disease is milder than NMOSD associated with AQP4-Ab. In this study, we describe the demographic characteristics, presentation, clinical and paraclinical features, and outcomes in the largest cohort of adult patients with TM who have tested positive for serum MOG-Ab to date, to our knowledge, and compare them with those of adult patients with TM and AQP4-Ab disease. In particular, we assess the features of the TM episode for factors associated with worse outcomes and increased relapse risk. With the understanding that MOG-Ab disease has been defined as a separate entity from AQP4-Ab disease^4^ and multiple sclerosis (MS), we aim to describe the characteristics associated with TM in this condition and highlight clinically relevant long-term outcomes.

## Methods

### Ethics

All patients included in this study signed written informed consent to be included in the Oxford NMOSD tissue bank, which was approved by the Oxford Research Ethics Committee. Enrolled participants signed consent for their clinical, imaging, and laboratory information, along with blood test results and questionnaire responses, to be used for research and publication. This study falls within this remit. The reporting of this research was done in conjunction with the Strengthening the Reporting of Observational Studies in Epidemiology (STROBE) reporting guideline for cross-sectional studies.

### MOG-Ab and AQP4-Ab Testing

Testing for MOG-Ab disease and AQP4-Ab disease was performed in the autoimmune neurology laboratory at the University of Oxford using cell-based assays as described previously.^5,6^ All included patients with MOG-Ab disease were found not to have AQP4-Ab disease and vice versa. In particular, the MOG-Ab assay was to full-length MOG with an IgG1-specific secondary antibody, which increases specificity.^5^

### Cohort

Every patient referred to our clinic is entered into a database using Access database software version 12.0 (Microsoft Corp) to facilitate patient care. At their first visit, each patient is offered the opportunity to sign written informed consent for their clinical data to be used for research. We screened the database for adult patients who had experienced a TM episode and had either MOG-Ab disease or AQP4-Ab disease from April 2018 to January 2019. Patients who had confounding neurological comorbidities, who had not signed consent, or for whom acute imaging or clinical details from the acute TM episode were unavailable were excluded. We collected demographic characteristics (ie, age at onset of disease, sex, and self-reported race/ethnicity), date of onset of the disease and date of last follow-up (used to calculate disease duration), features of the onset TM (ie, signs and symptoms), Expanded Disability Status Scale (EDSS) score at nadir, time to acute treatment and type of acute treatment, magnetic resonance image (MRI) features during acute episode, long-term disability outcomes (ie, motor and sphincter dysfunction), relapse history, long-term treatment, and features of any follow-up imaging performed outside of acute relapse treatment. Recovery from the onset episode of TM was described using a subgroup of patients who had been relapse free for at least 1 year after the TM episode to allow for full recovery to take place before the residual disability was measured.

Our service receives referrals from the entire southern United Kingdom, so MRI scans were performed locally during the acute TM episode and then reviewed at our center. All MRI scans were performed on 1.5-T or 3-T MRI machines and included sagittal cervical and thoracic T1 (slice thickness, 3-4 mm; echo time, 6.8-12.2 milliseconds; repetition time, 469-540 milliseconds) and T2 spinal cord imaging (slice thickness, 3-4 mm; echo time, 100-116 milliseconds; repetition time, 3000-4840 milliseconds). Not all participants were given contrast. Axial images acquired among these patients were either T2 or T2* weighted images in the affected area (slice thickness, 4-5 mm). These scans were then reviewed by one of us who specializes in inflammatory neuroradiology (W.K.) and 4 of us in the clinical team (R.M., S.M., M.I.L., and J.P.).

This study aimed to identify the features of TM in patients with MOG-Ab disease or AQP4-Ab disease during the acute episode and during follow up. We described and compared the clinical MRI features in each patient group during the TM episode. We then examined long-term outcomes of interest: disability, as measured by the EDSS, and the presence of long-term sphincter dysfunction. Finally, we aimed to assess the TM episode features that might be associated with a worse prognosis in each group. Owing to the differences in demographic characteristics that have been described in these conditions,^7^ we hypothesized that age and sex would be different between the groups and so were considered confounders. In all analyses, age, sex, and disease duration were included as variables.

### Statistical Analysis

Statistical analysis was performed using RStudio statistical software version 1.1.447 (RStudio) and Prism data visualization software version 6.0c (GraphPad). Mann-Whitney *U* tests were used when comparing continuous variables. Fisher exact test was used when comparing frequencies. The Kaplan-Meier method was used for estimating relapse risk and disability outcomes. There were no deaths in either group, and the time factor used in the Kaplan-Meier analysis was from the date of disease onset to the date of last follow-up. Binomial logistic and multivariate regression models were used to identify factors associated with relapse and disability.

Although age was used as a continuous variable in the regression analyses, for the purposes of the Kaplan-Meier analysis, age was dichotomized according to the median cutoff of 50 years (ie, aged <50 vs ≥50 years). In each regression analysis, a set of clinically relevant potentially associated factors were selected and each assessed with a univariate analysis. The significant factors were then entered into the multiple regression model with the factors that differed between MOG-Ab disease and AQP4-Ab disease groups (ie, sex, age, and disease duration). *P* values were 2-tailed, and statistical significance was set at .05. Data analysis was conducted from February 2019 to April 2019.

## Results

### Demographic Characteristics

Among 153 patients admitted to our clinic with MOG-Ab disease or AQP4-Ab disease who had experienced a TM episode, 5 were excluded for having confounding neurological comorbidities, 13 patients were excluded because they had not signed informed consent, and 10 were excluded because we were unable to track any acute imaging or clinical details from the acute TM episode. The final cohort included 115 patients, including 46 adult patients with MOG-Ab disease (mean [SD] age at disease onset, 33.7 [11.2] years; 24 [52%] women; 36 [78%] white race) and 69 adult patients with AQP4-Ab disease (mean [SD] age at disease onset, 48.5 [14.9] years; 57 [83%] women; 40 [58%] white race). The demographic characteristics of each group are presented in the Table. The differences in age, sex, and race/ethnicity were in keeping with the demographic distributions of groups in the literature (a higher proportion of women and nonwhite patients in the AQP4-Ab group).^8^ Median (range) disease duration, defined as time from the first episode of TM to the last clinical visit at which their condition could be verified, was longer in patients with AQP4-Ab disease (81.9 [3.9-297.3] months) than patients with MOG-Ab disease (28.5 [3.5-399.0] months). In both groups, it was more common for the first TM episode to occur at the onset of the disease (32 patients with MOG-Ab disease [70%]; 54 patients with AQP4-Ab disease [78%]).

**Table.** Demographic, Clinical, and Magnetic Resonance Imaging (MRI) Characteristics Among Patients With MOG-Ab Disease or AQP4-Ab Disease

Characteristic	Patients, No. (%)		*P* Value
	MOG-Ab Group (n = 46)	AQP4-Ab Group (n = 69)	
Sex			
Men	22 (48)	12 (17)	<.001
Women	24 (52)	57 (83)	
Age at disease onset, mean (SD), y	34 (11)	48 (15)	<.001
Race/ethnicity			
White	36 (78)	40 (58)	.03
East Asian	3 (7)	6 (9)	.74
South Asian	1 (2)	4 (6)	.65
Afro-Caribbean	0	16 (23)	<.001
Mixed	3 (7)	3 (4)	.68
Unknown	3 (7)	0	.06
Disease duration, median (range), mo	28.5 (3.5-399.0)	81.9 (3.9-287.3)	.003
First TM episode occurred at disease onset	32 (70)	54 (78)	.51
Concurrent symptoms present with TM			
ON	12 (26)	2 (3)	<.001
Brainstem manifestations	6 (13)	8 (12)	>.99
Brain manifestations	4 (9)	1 (2)	.16
Myelitis symptoms at onset			
Sensory only	9 (20)	6 (9)	.09
Motor only	2 (4)	0	.16
Pain	6 (13)	20 (29)	.04
Sphincter involvement	34 (74)	39 (57)	.08
EDSS score			
At nadir			
Mean (SD)	5.8 (2.1)	6.5 (2.0)	.07
Median (range)	5.5 (1.0-9.0)	7.0 (3.0-9.5)	
At recovery, median (range)	1.8 (1.0-8.0)	3.0 (1.0-8.0)	
At last follow-up			
Mean (SD)	2.0 (1.7)	4.1 (2.5)	<.001
Median (range)	2.0 (0-8.0)	4.0 (1.0-8.0)	
≥6	3 (7)	30 (44)	<.001
Long-term outcome			
Sphincter dysfunction	27 (59)	33 (48)	.34
Catheter usage	9 (20)	16 (23)	.81
Relapse after TM	17 (37)	36 (52)	
Time to relapse, mean (SD), mo	31 (34)	32 (33)	.87
Type of relapse			
ON	9 (53)	3 (8)	.012
TM	2 (12)	25 (69)	<.001
Simultaneous ON and TM	3 (18)	7 (19)	>.99
Brainstem manifestation	2 (12)	1 (3)	.24
Brain manifestation	1 (6)	0	.32
MRI features			
Spinal cord			
Short lesions only	11 (24)	8 (12)	.12
Long lesions only	24 (52)	59 (86)	<.001
Long and short lesions	11 (24)	2 (3)	<.001
Total lesion length, mean (SD), vertebral levels	6.8 (6.5)	7.7 (5.2)	.38
Single lesion	28 (61)	62 (90)	<.001
Multiple lesions	18 (39)	7 (10)	<.001
Conus involvement	18 (39)	8 (12)	.001
Contrast enhancement, No./total No. (%)	17/24 (71)	38/48 (79)	.55
Axial cord			
No.	16	24	
Central	12 (75)	17 (71)	>.99
Lateral	3 (19)	3 (13)	.67
Posterior	0	0	>.99
Anterior	1 (6)	4 (17)	.63
Brain			
No.	45	62	
Normal	19 (42)	47 (76)	<.001
Brain lesion	24 (53)	14 (23)	<.001
Brainstem lesion	11 (24)	11 (18)	.47

Abbreviation: AQP4, aquaporin-4; EDSS, Expanded Disability Status Scale; MOG, myelin oligodendrocyte glycoprotein; ON, optic neuritis; TM, transverse myelitis.

### Clinical Presentation and Baseline Features of First TM

The frequency of motor and sensory symptoms and sphincter involvement at nadir did not significantly differ. Patients with AQP4-Ab disease were more likely to have neuropathic pain (most commonly back pain) at onset than those with MOG-Ab disease (20 patients [29%] vs 6 patients [13%]; *P* = .04). Patients with MOG-Ab disease were more likely to have ON concurrent with the first TM episode than patients with AQP4-Ab disease (12 patients [26%] vs 2 patients [3%]). Throughout the course of the disease, patients with MOG-Ab disease were more likely to have involvement in other areas of the nervous system, with only 2 patients (4%) with MOG-Ab disease having isolated spinal cord involvement compared with 39 patients (56%) with AQP4-Ab disease (*P* < .001).

The mean (SD) EDSS score at nadir was not significantly different between groups (MOG-Ab: 5.8 [2.1]; AQP4-Ab: 6.5 [2.0]), regardless of whether the first TM episode occurred at disease onset or as a relapse. There was no significant difference in the proportion of brainstem or brain manifestations occurring together with the TM episode. Among patients with MOG-Ab disease, brain manifestations constituted an acute disseminated encephalomyelitis (ADEM)–like presentation that occurred in 4 patients (9%). This ADEM-like presentation did not occur in any of the patients with AQP4-Ab disease.

Among patients with brainstem lesions, 6 of 11 patients (55%) with MOG-Ab disease and 8 of 11 patients (73%) with AQP4-Ab disease had symptomatic lesions, but asymptomatic brainstem lesions occurred as well. In patients with AQP4-Ab disease, the predominant symptoms of brainstem involvement included nausea, vomiting, and hiccups. Other symptoms experienced were vertigo and dysphagia; and 2 patients with high cervical lesions contiguous with medullary lesions required ventilatory support. In patients with MOG-Ab disease, bulbar symptoms were the most commonly reported, and there were no reports of vomiting or hiccups. Four patients with MOG-Ab disease presented with brainstem lesions in the context of an ADEM-like presentation.

A minority of patients in either group were receiving background immunosuppressive therapy at the time of their first TM episode, even if it was not their first disease episode (8 patients [17%] with MOG-Ab disease and 6 patients [9%] with AQP4-Ab disease ). As it was often an isolated ON that preceded the TM episode, antibody testing was only performed for most patients at the time of the spinal cord presentation; thus, the diagnosis could not be made earlier. Higher EDSS score at nadir was associated with longer total lesion length in patients with MOG-Ab disease (*R*^2^ = 0.19; *P* = .006) and AQP4-Ab disease (*R*^2^ = 0.15; *P* = .003) .

### Spinal Cord MRI Features Within 4 Weeks of Symptom Onset

Short lesions (<3 vertebral levels) with no concomitant long lesion occurred in 11 patients (24%) with MOG-Ab disease and 8 patients (12%) with AQP4-Ab disease (Table). The presence of short lesions was particularly prevalent in patients with MOG-Ab disease whose first TM episode occurred as a relapse rather than at onset; 50% of these patients had only short lesions (7 of 14 patients) compared with 13% of those whose TM episode occurred at the onset of the disease (4 of 32 patients). This pattern did not occur in the AQP4-Ab group; the distribution of short lesions was 13% among patients who experienced the first TM episode at disease onset (7 of 54 patients) and 7% among patients who experienced the first TM episode at relapse (1 of 15 patients). Additionally, 11 patients (24%) with MOG-Ab disease presented with longitudinally extensive TM (LETM), defined as a lesion of 3 or more vertebral levels, with additional short lesions, whereas this occurred in only 2 patients (3%) with AQP4-Ab disease. The remaining 24 patients (52%) with MOG-Ab disease and 59 patients (86%) with AQP4-Ab disease presented with only LETM. Compared with patients with AQP4-Ab disease, patients with MOG-Ab disease were more likely to have multiple lesions (18 patients [39%] vs 7 patients [10%]; *P* < .001) and involvement of the conus (18 patients [39%] vs 8 patients [12%]; *P* < .001). Among 40 patients for whom axial MRI data were available, we did not find statistically significant differences in the axial location, and central lesions were the most common lesions in both groups (12 of 16 patients [75%] with MOG-Ab disease; 17 of 24 patients [71%] with AQP4-Ab disease) or in the frequency of gadolinium enhancement among 72 patients with acute contrast scan data from within the 4-week window that we considered the acute scan period (17 of 24 patients [71%] with MOG-Ab disease; 38 of 48 patients [79%] with AQP4-Ab disease). Of note, in patients for whom we had axial imaging results, 1 patient (7%) with MOG-Ab disease and 1 patient (4%) with AQP4-Ab disease presented with only short, lateral lesions, such as those typically seen in patients with MS.

### Acute Intracranial Imaging

#### Brain Imaging

Patients with MOG-Ab disease were more likely than patients with AQP4-Ab disease to have white matter lesions on brain MRI (24 patients [53%] vs 14 patients [23%]; *P* < .001). Among patients with MOG-Ab disease who had brain lesions (excluding brainstem lesions), 2 (8%) had nonspecific white matter lesions, 2 (8%) had MS-like lesions (ie, juxtacortical and periventricular), 4 (17%) had ADEM-like (ie, large and fluffy) lesions that were symptomatic, and 16 (67%) had 1 to 3 ADEM-like lesions that occurred in different locations and that did not correspond to any brain manifestations at presentation. Thus, in the total MOG-Ab group, 20 patients (44%) had ADEM-like lesions on the brain. There were no borderline cases classified as ADEM in which lesions may have been considered nonspecific white matter lesions. In patients with AQP4-Ab disease, it was more common to find brain lesions (excluding brainstem lesions) in older patients, making it difficult to differentiate such lesions from vascular changes associated with age in some patients. Lesions in the brain constituted mainly nonspecific white matter lesions (6 patients [43%]); the other lesions included 1 patient (7%) with cerebellar lesions, 2 patients (14%) with callosal lesions, and 1 patient (7%) with MS-like lesions (ie, periventricular white matter lesions), and the remaining 4 patients (29%) had inflammatory lesions typical for NMOSD associated with AQP4-Ab disease.

#### Brainstem Imaging

The presence of associated brainstem lesions was not significantly different between groups. However, when an associated brainstem lesion occurred at the time of a TM, it was more commonly contiguous with the cord lesion in patients with AQP4-Ab disease than patients with MOG-Ab disease (8 patients [72%] vs 3 patients [27%]; *P* = .08). Of 11 patients with MOG-Ab disease and brainstem lesions, 4 (36%) had an ADEM-like presentation at onset, including lesions in the brain and brainstem, 2 (18%) had lower brainstem involvement contiguous with an upper cervical cord lesion, 1 (9%) had a lesion in the medulla that did not extend into the cervical cord, 1 (27%) had a midbrain lesion (9%), and 3 had lesions in the pons.

### Cerebrospinal Fluid Features

Of 62 cerebrospinal fluid samples available (25 from the MOG-Ab group and 37 from the AQP4-Ab group), there was no significant difference in cerebrospinal fluid findings between the 2 groups. White blood cell counts were often increased (range: 10-220 per μL [to convert to ×10^9^ per liter, multiply by 0.001] in patients with MOG-Ab disease; 14-162 per μL in patients with AQP4-Ab disease). Total cerebrospinal fluid protein was also commonly increased (range: 55-190 g/dL [to convert to grams per liter, multiply by 10] in patients with MOG-Ab disease; 50-159 g/dL in patients with AQP4-Ab disease). Tests for oligoclonal bands were positive for 5 patients (20%) with MOG-Ab disease and 6 patients (17%) with AQP4-Ab disease.

### Recovery From First TM Episode Among Patients With a Single TM Episode and No Relapse for at Least 1 Year

The subgroups of patients who experienced only 1 TM episode and no relapse for at least 1 year included 29 patients with MOG-Ab disease (mean [SD] age, 37.0 [12.1] years; 16 [55%] women) and 44 patients with AQP4-Ab disease (mean [SD] age, 52.6 [14.8] years; 37 [84%] women). The median (range) recovery EDSS score was significantly lower in patients with MOG-Ab disease compared with patients with AQP4-Ab disease (1.8 [1.0-8.0] vs 3.0 [1.0-8.0]; *P* < .001). Despite better motor outcomes among patients with MOG-Ab disease, persistent bladder dysfunction (eg, urgency, hesitancy, incontinence, frequent urinary tract infections) was more common among patients with MOG-Ab disease than patients with AQP4-Ab disease (17 patients [59%] vs 21 patients [48%]). The proportion of patients who required long-term intermittent or in situ catheterization was roughly equal (6 patients [24%] with MOG-Ab disease vs 11 patients [25%] with AQP4-Ab disease). Persistent bowel symptoms (eg, constipation or incontinence) occurred in 11 patients (38%) with MOG-Ab disease and 18 patients (41%) with AQP4-Ab disease. Among men, erectile dysfunction more commonly occurred in patients with MOG-Ab disease than those with AQP4-Ab disease (6 patients [46%] vs 2 patients [29%]; *P* < .001).

### Relapses

In 14 patients (30%) with MOG-Ab disease and 15 patients (22%) with AQP4-Ab disease, the index TM occurred after 1 or more previous non-TM episode. Seventeen patients (37%) with MOG-Ab disease and 36 patients (52%) with AQP4-Ab disease went on to have a relapse after the first TM episode; follow-up times were comparable between groups. At the time of the relapse, 2 patients with MOG-Ab disease (12%) and 21 patients with AQP4-Ab disease (58%) were receiving immunosuppressive treatment (among patients with AQP4-Ab disease, this meant an immunosuppressive medication in addition to oral prednisolone). An additional 2 patients (12%) with MOG-Ab disease had been undergoing immunosuppressive treatment for at least 6 months after their onset TM episode but then went on to relapse after the treatment window. There was no significant difference in the mean time to relapse between the 2 groups.

Among patients with MOG-Ab disease, 9 (53%) relapsed with ON after a TM episode, 3 (18%) relapsed with ON and TM, 2 (12%) experienced another episode of TM, and 3 (18%) experienced brainstem or brain manifestations in relapse. Among patients with AQP4-Ab disease, 25 (69%) relapsed with another episode of TM, 7 (19%) relapsed with ON and TM, 3 (8%) relapsed with ON, and 1 (3%) experienced brainstem manifestations in relapse. This was also statistically significantly different when comparing the 2 groups (*P* < .001).

**Figure 1.**
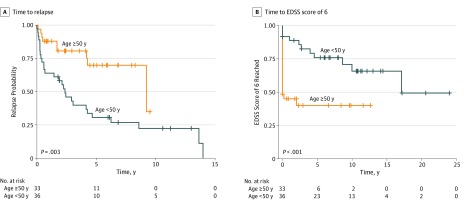
Kaplan-Meier Analysis of Patients With Aquaporin-4 Antibody Disease Stratified by Age Crosses indicate censored patients; EDSS, Expanded Disability Status Scale.

In patients with AQP4-Ab disease, age was associated with relapse risk, with younger patients more at risk (odds ratio, 0.94 [95% CI, 0.88-0.98]; *P* < .001). Disease duration was also associated with risk of relapse (odds ratio, 1.02 [95% CI, 1.01-1.04]; *P* = .02). There were no associations of treatment, sex, or onset severity with risk of relapse. To further explore the association of age with relapse, a survival analysis was performed. Using the median cutoff of age 50 years, patients in the younger group (n = 36) were more likely to relapse than those in the older group (n = 33) (log-rank: *P* = .003) (Figure 1A). The mean disease duration did not differ between the older and younger groups.

Among patients with MOG-Ab disease, there was no specific characteristic associated with relapses, and the only statistically significant factor was disease duration (odds ratio, 1.002 [95% CI, 1.001-1.004]; *P* = .004). The association of age with relapse observed in patients with AQP4-Ab disease was not observed in those with MOG-Ab disease.

### Disability

In patients with AQP4-Ab disease, the factors associated with EDSS score in the univariate analysis were age and severity of the onset episode, and they retained statistical significance in a multivariate analysis (*R*^2^ = 0.67; *P* = .003). In a survival analysis using the 2 age groups, the younger group, although more likely to relapse, was less likely to reach an EDSS score of 6 (log-rank: *P* < .001) (Figure 1B).

**Figure 2.**
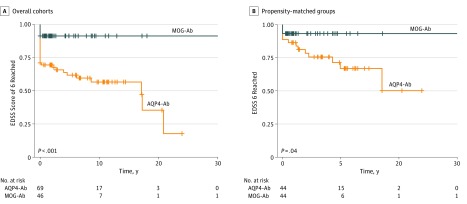
Kaplan-Meier Analysis of Time to an EDSS Score of 6 in Patients With MOG-Ab Disease or AQP4-Ab Disease Crosses indicate censored patients; Ab, antibody; AQP4, aquaporin-4; EDSS, Expanded Disability Status Scale; and MOG, myelin oligodendrocyte glycoprotein.

Compared with patients with MOG-Ab disease, more patients with AQP4-Ab disease reached an EDSS score of 6 after their first TM episode (3 patients [7%] vs 30 patients [44%]; *P* < .001). The difference remained statistically significant in the Kaplan-Meier analysis of patients with AQP4-Ab disease compared with the total cohort at last follow-up (log-rank: *P* < .001) (Figure 2A).

Because of the association of age with disability in patients with AQP4-Ab disease, and acknowledging that the mean age of this group was older, we used the nearest neighbor method in propensity score–matching to obtain 2 more closely matched subgroups. These consisted of 44 patients with MOG-Ab disease (mean [SD] age, 34.4 [10.9] years; 24 [55%] women) and 44 patients with AQP4-Ab disease (mean [SD] age, 38.9 [10.9] years; 32 [73%] women). We then performed a Kaplan-Meier analysis on these groups, and patients with AQP4-Ab disease were still more likely to reach an EDSS score of 6 (Figure 2B).

**Figure 3.**
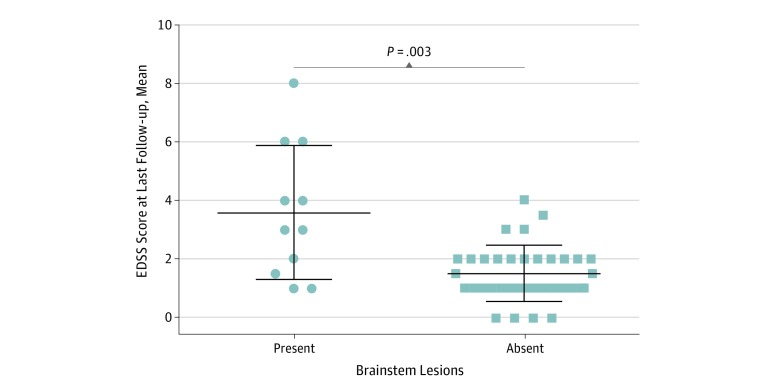
Mean EDSS Score at Last Follow-up in Patients With Myelin Oligodendrocyte Glycoprotein–Antibody Disease Stratified by Presence of Brainstem Lesions at the Time of Transverse Myeltis Episode Center lines indicate means; upper and lower lines, SDs. EDSS indicates Expanded Disability Status Scale.

Among patients with MOG-Ab disease, higher EDSS score was associated with the presence of a brainstem lesion, whether symptomatic or asymptomatic (*R*^2^ = 0.34; *P* < .001), but not with age, sex, the presence of brain lesions, disease duration, or EDSS score at nadir. The mean (SD) long-term EDSS score in patients with brainstem lesions in MRI results was significantly higher than in patients without brainstem lesions (3.5 [2.3] vs 1.4 [0.9]; *P* < .001), regardless of whether the lesion occurred at onset or as a relapse (Figure 3). Median (range) disease duration between the 2 groups was not significantly different (patients with brainstem lesions, 38 [14-206] months; patients without brainstem lesions, 26 [4-399] months).

### Long-term Sphincter Dysfunction

There was no significant difference between patients with MOG-Ab disease and patients with AQP4-Ab disease in residual sphincter dysfunction (27 patients [59%] with MOG-Ab disease vs 33 patients [48%] with AQP4-Ab disease) or in the need for long-term catheterization (9 patients [20%] with MOG-Ab disease vs 16 patients [23%] with AQP4-Ab disease).

**Figure 4.**
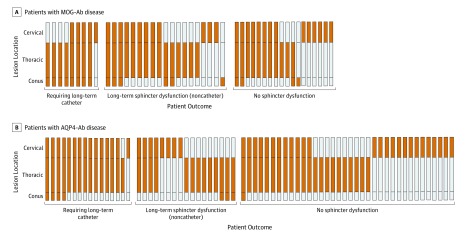
Sagittal Location of Lesions and Sphincter Dysfunction at Last Follow-up in Patients With Myelin Oligodendrocyte Glycoprotein (MOG) Antibody (Ab) Disease or Aquaporin-4 (AQP4) Ab Disease

Among patients with MOG-Ab disease, the factor associated with need for long-term catheterization was the presence of a conus lesion (*R*^2^ = 0.33; *P* = .002). The need for long-term catherization was not associated with the severity of the episode or EDSS score at last follow-up. Among patients with AQP4-Ab disease, the univariate analysis suggested that the need for long-term catheterization was associated with EDSS score at last follow up (*R*^2^ = 0.19; *P* = .02) and the presence of a conus lesion (*R*^2^ = 0.19; *P* = .048). Figure 4 presents the locations of lesions in patients who required long-term catheterization, patients who had sphincter dysfunction without requiring catheterization, and unaffected patients in each group. Sphincter dysfunction existed as a spectrum in both conditions, from mild to requiring catheterization.

In the recovery analysis, erectile dysfunction occurred more commonly in patients with MOG-Ab disease than in those with AQP4-Ab disease (9 men [41%] vs 1 man [8%]; *P* = .52). This difference persisted to the increased presence of erectile dysfunction at last follow-up in patients with MOG-Ab disease.

### Follow-up MRI

Among 67 patients with follow-up MRI results outside of an acute relapse, there were 26 patients (57%) with MOG-Ab disease and 41 patients (59%) with AQP4-Ab disease. These scans were conducted at least 6 months after an acute relapse. Complete resolution of abnormalities in MRI findings, without atrophy, occurred in 16 patients (62%) with MOG-Ab disease and 7 patients (17%) with AQP4-Ab disease (*P* < .001).

## Discussion

In this study, we describe the largest single-center longitudinal cohort, to our knowledge, of adult patients with MOG-Ab disease who experienced at least 1 TM episode and compared their outcomes with those of patients with AQP4-Ab disease who experienced at least 1 TM episode. In particular, we highlighted the outcomes and prognostic features in each group. We found that in patients with MOG-Ab disease, short spinal cord lesions and multiple lesions at disease onset were more common, which suggests that these patients may be at higher risk of receiving a misdiagnosis of MS. This is of particular importance in patients with MOG-Ab disease who have their first TM as a relapse, among whom approximately half presented with only short lesions on spinal cord MRI results. Brain lesions also occurred more commonly in patients with MOG-Ab disease at the time of their TM episode and were often asymptomatic. In particular, we found that the presence of a brainstem lesion at the time of a TM episode in patients with MOG-Ab disease may be a risk factor for a worse recovery. In patients with AQP4-Ab disease, the overall disability at last follow-up was greater with older age at disease onset and was associated with a worse recovery, and a younger age at disease onset was associated with a higher relapse risk.

There were noticeable differences in age and sex between the 2 groups. We used propensity score matching to analyze a more closely matched subgroup; however, owing to our AQP4-Ab group having significantly fewer men, it was difficult to fully match the groups without removing all men from the analysis. While all results should be considered in light of the age and sex differences, AQP4-Ab disease appeared to be associated with worse overall disability.

In both groups, TM was more likely to occur at disease onset. A 2013 study^9^ found that up to 47% of patients with AQP4-Ab disease went on to have disease limited to the spinal cord (either monophasic or recurrent TM). Our results were in keeping with this finding in that 56% of patients with AQP4-Ab disease had disease isolated to the spinal cord. However, in patients with MOG-Ab disease, this was far less likely, as only 2 patients (4%) had isolated cord involvement in the course of their disease. Nearly 70% of patients with AQP4-Ab disease who went on to relapse experienced repeated episodes of TM; however, repeated TM episodes occurred in only 12% of patients with MOG-Ab disease, and they were more likely to relapse with ON. Therefore, patients with relapsing MOG-Ab disease were more likely to fulfill the 2015 NMOSD diagnostic criteria^10^ for AQP4-Ab–negative NMOSD at follow-up. Our study supports the findings of a 2014 comparison study^7^ of these 2 conditions that recommended testing all such patients for MOG-Ab disease.

Previous smaller studies in AQP4-Ab disease have highlighted the significance in the length of the lesion at onset.^11,12^ In this study, the length of the lesion was associated with the severity of the disease onset presentation in both groups. In patients with AQP4-Ab disease, older age at onset was associated with the long-term disability outcome of reaching an EDSS score of 6. A 2016 study^13^ found an association of age with relapses in patients with AQP4-Ab disease, and our study also found that in patients with AQP4-Ab disease who experienced a TM episode, the younger age group was more likely to relapse; older patients were less likely to relapse but accumulated disability from the onset episode. In patients with MOG-Ab disease, residual disability was accumulated from the onset episode regardless of age at onset.

Important information was obtained from MRI findings in these conditions. We found that up to 48% of patients with MOG-Ab disease who experienced a TM episode presented with short lesions at onset, and 24% presented with only short lesions on their initial MRI results and no associated LETM. We also found that patients with MOG-Ab disease more commonly presented with multiple spinal cord lesions than patients with AQP4-Ab disease did, which agrees with results presented in a 2019 study by Dubey et al.^14^ Short cord lesions may be misdiagnosed as MS, particularly in pediatric populations, in which patients with MOG-Ab disease have received an MS misdiagnosis in the past.^15^ Therefore, axial imaging of the spinal cord is crucial in these cases, as is brain imaging. In our adult-only cohort, brain lesions occurred in more than half of patients with MOG-Ab disease at the time of the first TM episode, and most lesions were asymptomatic. Of note, 41% of patients with MOG-Ab disease had ADEM-like brain lesions, whereas none of the patients with AQP4-Ab disease had ADEM-like brain lesions. Therefore, ADEM-like lesions occurring together with an LETM or concomitant ON (unilateral or bilateral) may be an indicator for MOG-Ab disease.

This study found that involvement of the brainstem at the time of the TM, whether symptomatic or asymptomatic, was associated with worse outcomes in patients MOG-Ab disease. The mean EDSS score at recovery in patients with MOG-Ab disease with brainstem lesions was significantly higher than in those without brainstem lesions. To our knowledge, this is the first study that found this potentially important association. This association was not observed in patients with AQP4-Ab disease.

The predilection for MOG-Ab disease to involve the conus has been suggested as an important differentiator between MOG-Ab disease and AQP4-Ab disease.^3,7,14,16,17^ The prevalence of conus involvement in our study in patients with MOG-Ab disease was 39%, and although prior reports have varied from 4%^18^ to 75%,^19^ a 2019 study by Dubey et al^14^ aligned with our findings. We also found that involvement of the conus was associated with long-term sphincter dysfunction, particularly the requirement for catheterization. While sphincter dysfunction is not limited to patients with MOG-Ab disease, the proportion of patients with long-term sphincter dysfunction was not proportional to the recovery of mobility compared with patients with AQP4-Ab disease. Therefore, as an outcome measure, the EDSS score, which is primarily a measure of mobility, did not fully address the outcomes in MOG-Ab disease, in which mobility may be recovered but sphincter dysfunction remains an important outcome that needs to be quantified and treated effectively, as it significantly affects quality of life.^20^ When examining the lesion locations in patients requiring a catheter, we hypothesize that sphincter dysfunction occurs by 1 of 2 mechanisms: (1) severe involvement of the spinal cord above the conus, such as that seen in AQP4-Ab disease, is generally more destructive and more likely to affect mobility, or (2) damage to the conus itself, which may be more vulnerable even with a less severe underlying disease, occurs more commonly in patients with MOG-Ab disease.

A 2017 study^21^ of follow-up brain MRIs outside of relapse suggested resolution of lesions was more common in MOG-Ab disease. Our study’s findings in the spinal cord were consistent with this. Up to 62% of patients with MOG-Ab disease showed complete resolution of lesions in MRI findings at least 6 months after the first TM episode. This occurred in only 17% of patients with AQP4-Ab disease, in whom residual lesions or atrophy were more common. Studies using nonconventional MRI methods to examine the integrity of the normal-appearing cord after an episode of TM are needed to establish if this recovery is full. The pathological basis for these differences in clinical and MRI features should also be further explored.

### Limitations and Strengths

Our study had limitations. One limitation was that we used retrospective data collection. Additionally, MRI scans were from different centers, so not all patients were given contrast or had axial views taken. However, a strength of this study was that these data were more relevant to clinical practice with the observations having been made in a realistic clinical setting and patients having been scanned at different centers. Another limitation was that this study had small sample sizes, so variable selection for multivariate models may be misleading.^22^ However, we selected clinically relevant confounders and included potential variables, namely age, sex, and disease duration.

## Conclusions

In conclusion, the differences this study found between patients with MOG-Ab disease and patients with AQP4-Ab disease may assist clinicians in better risk stratification. Patients with MOG-Ab disease who experienced a TM episode were likely to fulfill criteria for Ab-negative NMOSD, so MOG-Ab disease should always be screened for. This study also found that short cord lesions were common in patients with MOG-Ab disease, particularly at the time of a TM relapse. Additionally, the differences between MOG-Ab disease and AQP4-Ab disease suggest that long-term disability outcomes are different in each group, and treatment response should be defined by disease-specific outcome measures.

## References

[1] Jarius S, Wildemann B AQP4 antibodies in neuromyelitis optica: diagnostic and pathogenetic relevance. Nat Rev Neurol. 2010;6(7):-. doi:10.1038/nrneurol.2010.72 20639914

[2] Reindl M, Di Pauli F, Rostásy K, Berger T The spectrum of MOG autoantibody-associated demyelinating diseases. Nat Rev Neurol. 2013;9(8):455-461. doi:10.1038/nrneurol.2013.118 23797245

[3] Jurynczyk M, Messina S, Woodhall MR, Clinical presentation and prognosis in MOG-antibody disease: a UK study. Brain. 2017;140(12):3128-3138. doi:10.1093/brain/awx276 29136091

[4] Weber MS, Derfuss T, Metz I, Brück W Defining distinct features of anti-MOG antibody associated central nervous system demyelination. Ther Adv Neurol Disord. 2018;11:1756286418762083. doi:10.1177/1756286418762083 29623106 PMC5881972

[5] Waters P, Woodhall M, O’Connor KC, MOG cell-based assay detects non-MS patients with inflammatory neurologic disease. Neurol Neuroimmunol Neuroinflamm. 2015;2(3):e89. doi:10.1212/NXI.0000000000000089 25821844 PMC4370386

[6] Waters PJ, Pittock SJ, Bennett JL, Jarius S, Weinshenker BG, Wingerchuk DM Evaluation of aquaporin-4 antibody assays. Clin Exp Neuroimmunol. 2014;5(3):290-303. doi:10.1111/cen3.12107 27840658 PMC5102503

[7] Kitley J, Waters P, Woodhall M, Neuromyelitis optica spectrum disorders with aquaporin-4 and myelin-oligodendrocyte glycoprotein antibodies: a comparative study. JAMA Neurol. 2014;71(3):276-283. doi:10.1001/jamaneurol.2013.5857 24425068

[8] Sato DK, Callegaro D, Lana-Peixoto MA, Distinction between MOG antibody-positive and AQP4 antibody-positive NMO spectrum disorders. Neurology. 2014;82(6):474-481. doi:10.1212/WNL.0000000000000101 24415568 PMC3937859

[9] Kitley J, Leite MI, Küker W, Longitudinally extensive transverse myelitis with and without aquaporin 4 antibodies. JAMA Neurol. 2013;70(11):1375-1381. doi:10.1001/jamaneurol.2013.3890 23999580

[10] Tan CT, Mao Z, Qiu W, Hu X, Wingerchuk DM, Weinshenker BG International consensus diagnostic criteria for neuromyelitis optica spectrum disorders. Neurology. 2016;86(5):491-492. doi:10.1212/WNL.0000000000002366 26833940

[11] Murchison A, Kitley J, Leite MI, Küker W, Palace J Predictive value of MRI parameters in severity and recovery of first-episode myelitis in aquaporin-4 antibody disease. J Neurol Sci. 2015;355(1-2):49-53. doi:10.1016/j.jns.2015.05.011 26026944

[12] Mealy MA, Mossburg SE, Kim SH, Long-term disability in neuromyelitis optica spectrum disorder with a history of myelitis is associated with age at onset, delay in diagnosis/preventive treatment, MRI lesion length and presence of symptomatic brain lesions. Mult Scler Relat Disord. 2019;28(28):64-68. doi:10.1016/j.msard.2018.12.011 30554040 PMC6397677

[13] Tackley G, O’Brien F, Rocha J, Neuromyelitis optica relapses: race and rate, immunosuppression and impairment. Mult Scler Relat Disord. 2016;7:21-25. doi:10.1016/j.msard.2016.02.014 27237752

[14] Dubey D, Pittock SJ, Krecke KN, Clinical, radiologic, and prognostic features of myelitis associated with myelin oligodendrocyte glycoprotein autoantibody. JAMA Neurol. 2019;76(3):301-309. doi:10.1001/jamaneurol.2018.4053 30575890 PMC6440233

[15] Hacohen Y, Mankad K, Chong WK, Diagnostic algorithm for relapsing demyelinating syndromes of the CNS in children. Lancet. 2017;389(S41). doi:10.1016/S0140-6736(17)30437-3 28615429

[16] Mariano R, Flanagan EP, Weinshenker BG, Palace J A practical approach to the diagnosis of spinal cord lesions. Pract Neurol. 2018;18(3):187-200. doi:10.1136/practneurol-2017-001845 29500319

[17] Ciccarelli O, Cohen JA, Reingold SC, Weinshenker BG; International Conference on Spinal Cord Involvement and Imaging in Multiple Sclerosis and Neuromyelitis Optica Spectrum Disorders Spinal cord involvement in multiple sclerosis and neuromyelitis optica spectrum disorders. Lancet Neurol. 2019;18(2):185-197. doi:10.1016/S1474-4422(18)30460-5 30663608

[18] Kitley J, Woodhall M, Waters P, Myelin-oligodendrocyte glycoprotein antibodies in adults with a neuromyelitis optica phenotype. Neurology. 2012;79(12):1273-1277. doi:10.1212/WNL.0b013e31826aac4e 22914827

[19] Jarius S, Ruprecht K, Kleiter I, ; Neuromyelitis Optica Study Group MOG-IgG in NMO and related disorders: a multicenter study of 50 patients, part 2: epidemiology, clinical presentation, radiological and laboratory features, treatment responses, and long-term outcome. J Neuroinflammation. 2016;13(1):280. doi:10.1186/s12974-016-0718-0 27793206 PMC5086042

[20] Fumincelli L, Mazzo A, Martins JCA, Henriques FMD, Orlandin L Quality of life of patients using intermittent urinary catheterization. Rev Lat Am Enfermagem. 2017;25(0):e2906. doi:10.1590/1518-8345.1816.2906 28699993 PMC5511000

[21] Jurynczyk M, Geraldes R, Probert F, Distinct brain imaging characteristics of autoantibody-mediated CNS conditions and multiple sclerosis. Brain. 2017;140(3):617-627. doi:10.1093/brain/aww350 28364548

[22] Heinze G, Dunkler D Five myths about variable selection. Transpl Int. 2017;30(1):6-10. doi:10.1111/tri.12895 27896874

